# Chemical Aspects of Gut Metabolism of Flavonoids

**DOI:** 10.3390/metabo9070136

**Published:** 2019-07-10

**Authors:** Jaehong Han

**Affiliations:** Metalloenzyme Research Group and Department of Plant Science and Technology, Chung-Ang University, Anseong 17546, Korea; jaehongh@cau.ac.kr

**Keywords:** gut metabolism, flavonoids, demethylation, *C*-glucoside, natural products

## Abstract

The intestine is a small world where all the chemical reactions are operated by gut microbiota. Study on the gut metabolism of natural products is a new and expanding research area that leads to new bioactive metabolites, as well as novel chemical reactions. To provide exemplary cases, flavonoid biotransformation by intestinal bacteria with focus on *S*-equol biosynthesis and aryl methyl ether cleavage reaction, is described in this review.

## 1. Introduction

**Figure 1:**
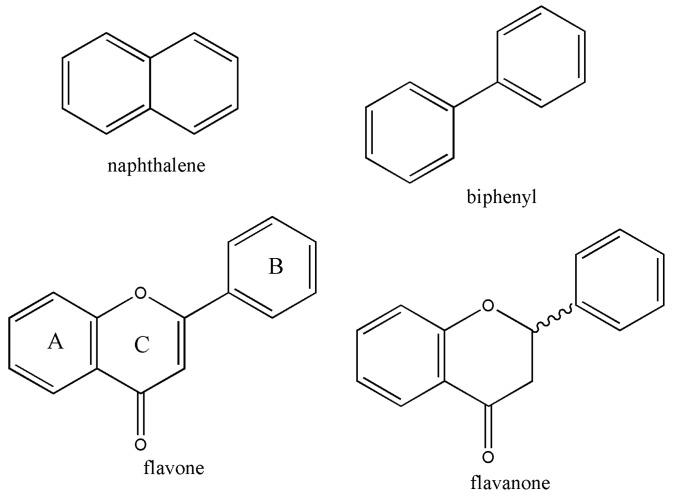
Molecular structures of flavone and the related compounds.

Study on the intestinal metabolism of natural products is an emerging research topic in natural product-related research. Besides, gut metabolism of natural products is a missing link explaining the sometimes unexplainable biological activity of bioactive natural compounds [1]. As a multidisciplinary study, it can be applied to clinical nutrition, pharmacokinetics, animal feeds and chemical biology. Chemical study on gut metabolism of natural products was initiated by *S*-equol, which was then known as a phytoestrogenic metabolite only produced by intestinal metabolism. The screening of *S*-equol-producing bacteria was first pioneered by Hor-Gil Hur, who had been working on the environmental microbiology for the bioremediation of non-degradable small organic halogens [2]. One of his research interests was related to the well-known bacterial biotransformation of naphthalene and biphenyl catalyzed by non-heme Fe-oxygenase, and flavone was chosen due to its structural similarity (Figure 1). Chromone ring of flavone, A and C rings, resembles naphthalene, and the connected B and C rings of flavone resemble biphenyl structure.

The non-heme Fe-oxygenase system is responsible for the aerobic biotransformation of most aromatic compounds and initiates mineralization of these environmentally persistent pollutants. Interestingly, epoxide formation was observed when flavanone, of which B-ring is free to rotate, was reacted with biphenyl dioxygenase over-expressed from *Escherichia coli*. Previously, epoxide formation was a characteristic feature of aromatic oxygenation by heme oxygenases [3], and flavanone 2’,3’-epoxide formation was the first epoxide formation by non-heme Fe-dioxygenase. Flavanone epoxide formation was explained as the steric controlled oxygen transfer by Fe^V^=O(OH) reactive oxygen species [4].

After finding the new chemical conversion of flavonoids by Fe oxygenases, we paid our attention to the intestinal metabolism of flavonoids, because gut microbiota is a unique anoxic environment after all. In comparison to the flavonoids metabolism under the oxidative environment, it was thought the anaerobic metabolism of flavonoids could be a widow’s cruse which provides new chemical conversions.

**Figure 2:**
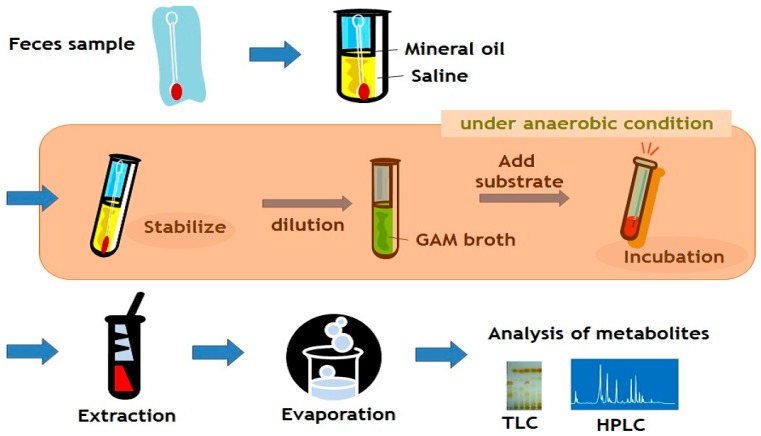
Experimental procedure for the screening of gut bacterium.

Experimentally, a single intestinal bacterium converting flavonoids with a selected functional group is screened and isolated, while maintaining the flavonoid biotransformation activity under the anaerobic conditions (Figure 2). The successful study depends on the efficient screening of bacteria, which requires anaerobic manipulation, as well as quick and efficient analysis of products. For the metabolism of flavonoids, thin layer chromatography (TLC) was often adopted. When the biotransformation of bioactive flavonoids occurs, it would lead to the new exciting chemistry, pharmacology, clinical nutrition and more. Furthermore, study on the metabolism can result in new bioactive metabolites, as well as new chemical reactions.

## 2. *S*-Equol Biosynthesis

**Figure 3:**
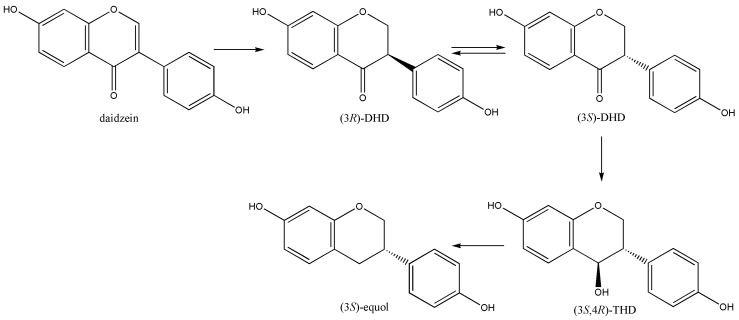
Stereospecific *S*-equol biosynthetic pathway.

The first *S*-equol producing bacterium, Julong-732, was isolated under Hur’s initiative at GIST, Korea [5,6], and *S*-equol biosynthetic pathway from daidzein was investigated by us [7]. The strict anaerobic bacterium *Eggerthella* sp. Julong-732 was isolated from the fecal sample of female volunteer, and showed the *S*-equol production only in GAM (Gifu Anaerobic Medium). Apparently, *S*-equol was a reduction product of daidzein with putative metabolic intermediates of dihydrodaidzein (DHD), tetrahydrodaidzein (THD), and dehydroequol (DE), taken from the resemblance of fatty acid biosynthetic pathway. However, dehydration of THD to DE was not observed and *S*-equol was not produced from DE either. Therefore, stereospecific conversion of daidzein to *S*-equol biosynthesis could not be explained by primary biochemical metabolism. Identification of metabolic intermediates DHD and THD, as well as their stereochemistry, was studied [8,9]. For the unique THD reductase, deuterium-labeled THD was synthesized and four stereoisomers were separated by chiral preparative HPLC to determine the absolute configuration [10]. In conclusion, achiral daidzein is reduced to *R*-DHD which is quickly racemized to *S*-DHD in aqueous solution (Figure 3). Then, *S*-DHD was further reduced stereospecifically to (3*S*, 4*R*)-THD, which is subsequently converted to *S*-equol [11].

Chemically, the reaction of THD to *S*-equol is of particular significance because stereospecific deoxygenation of the 4-OH group is precedented by ribonucleotide reductase. We proposed a radical reaction mechanism for *S*-equol production based on the isotope-labeled substrate conversion, and which was later confirmed by others [12]. Recently, the detail reaction mechanism and other equol producing THD reductase have been reviewed in depth by others [13]. Interestingly, when the substrate analogue of (3*S*,4*R*)-isoflavan-4-ol, (3*S*,4*R*)-THD without the 4’,7-dihydroxyl groups, was reacted with the THD reductase under the same reaction conditions, the expected reduction product of (3*S*)-isoflavan was not isolated. Therefore, it was suggested the two OH groups are important for the resonance stabilization of the reaction intermediate.

Encouraged by the newly found radical enzyme reaction of THD reductase, we have initiated research project of intestinal metabolism of flavonoids with the emphasis on the discovery of new biochemical reactions, such as aryl methyl ether cleavage.

## 3. Aryl Methyl Ether Cleavage

**Figure 4:**
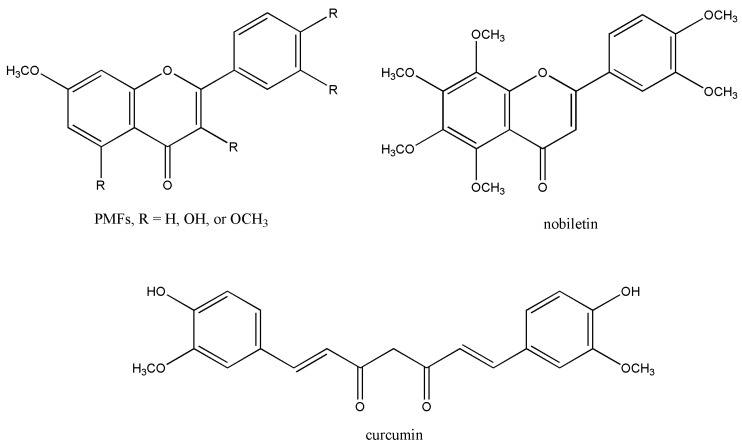
Molecular structures of polymethoxyflavones (PMFs) and other related natural compounds.

Polymethoxyflavones (PMFs) are flavonoids with several methoxy groups on the basic flavone skeleton. It is generally found from citrus plants and reported to exhibit a wide range of biological activity. Rhizomes of *Kaempferia parviflora* cultivated in Thailand and Laos as an ethnic herbal plant contain more than dozen of PMF analogs (Figure 4) [14]. Regardless of important biological activities, hydrophobicity of most PMFs in *K. parviflora* results in poor bioavailability. Demethylation of PMF, aryl methyl ether cleave, was expected to produce hydroxylflavones with different biological activity and enhanced water solubility.

**Figure 5:**
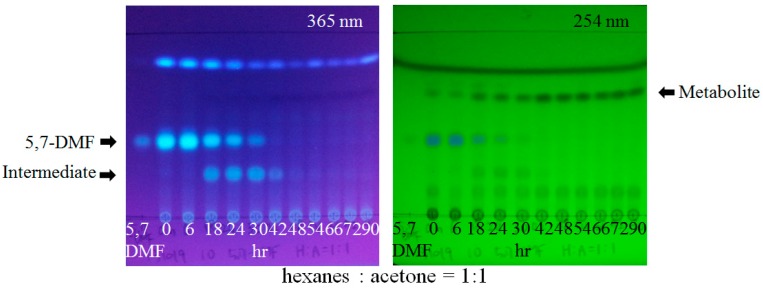
TLC analysis of the metabolites by *Blautia* sp. MRG-MPF1. The substrate, 5,7-dimethoxyflavone (5,7-DMF), was converted to metabolite, chrysin, via the intermediate later identified as 7-hydroxy-5-methoxyflavone.

After repetitive screening, 5,7-dimethoxyflavone-metabolizing *Blautia* sp. MRG-PMF1 was isolated (Figure 5) [15]. MRG-PMF1 was able to metabolize all the PMFs, including those isolated from *K. parviflora*. The largest substrate for this demethylation was nobiletin among the reacted substrates.

Chemically, aryl methyl ether cleavage requires strong Lewis acid, such as BBr_3_, that polarizes ether group which allows subsequent hydrolysis by water. Based on the experiments run with H_2_^18^O or ^13^CH_3_O-labeled substrate, it was found that the bioconversion of PMF is not a hydrolysis. In the meantime, substrate spectrum for the demethylation by *Blautia* sp. MRG-PMF1 was studied. It showed very vigorous demethylation for the aryl methyl ether compounds, from anisole to curcumin. Through the reactivity study and bioinformatics, the involvement of cobalamin-dependent enzymes was proposed [16].

## 4. Gut Metabolism to be Explored

**Figure 6:**
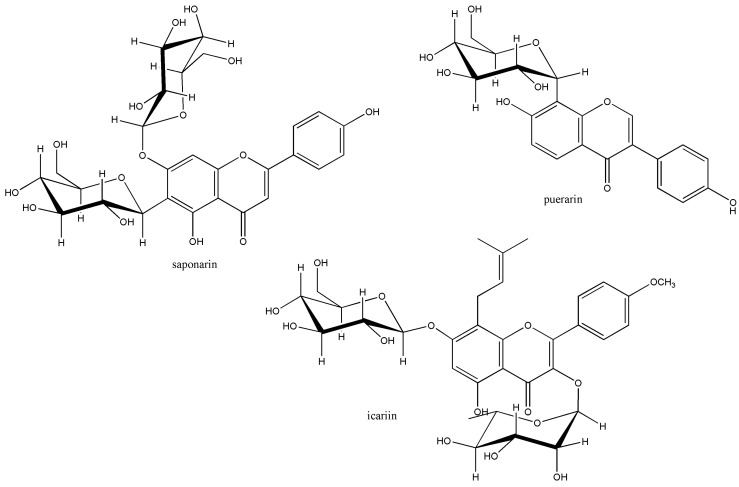
Molecular structures of selected *C*-glucoside and prenylated flavonoids.

Other interesting gut metabolisms, along with the aryl methyl ether cleavage, are C–C bond cleavage of *C*-glucosides and metabolism of prenylflavonoids (Figure 6). Gut metabolism of these natural products are less studied and expected to be difficult biochemical reactions. For example, *C*-glucoside flavonoids, including saponarin, are expected to interfere glucose metabolism as potential anti-diabetic treatment. Besides, *C*-glucoside cleavage with puerarin produces daidzein, which could lead to *S*-equol production from gut metabolism [17]. However, chemical mechanism for C–C bond cleavage for *C*-glucoside is not rationalized yet.

Due to the prenyl group which increases the hydrophobicity, prenylflavonoids are expected to have a unique and strong biological activity. But, the gut metabolism of prenylflavonoids was less studied. Recently, icariin, a prenylated kaempferol with methyl aryl ether and *O*-glycoside groups, was reported to be metabolized to demethylicaritin by human gut bacteria [18]. However, gut metabolism of prenylflavonoids has not been reported yet, even though prenyl group removal by C–C bond cleavage of 8-prenylnaringenin was reported from the pharmacokinetics study [19].

## 5. Conclusions

New discovery in environmental microbiology always leads to the emergence of new chemistry, especially bioinorganic chemistry. As a unique microenvironment, gut metabolism of natural products will yield exciting chemical reactions too. As we confirmed from the biosynthesis of *S*-equol and demethylation of PMF, chemical study on gut metabolism will expand our knowledge in biochemistry and natural products. It should be mentioned that omics study on gut microorganism cannot answer every metabolic feature of gut metabolism. For example, we have found that the same bacteria, identified by 16S rDNA sequences, showed different activity of deglycosylation of flavonoids.
